# Comparison between Two-Dimensional and Point Shear Wave Elastography Techniques in Evaluating Liver Fibrosis Using Histological Staging as the Reference Standard: A Prospective Pilot Study

**DOI:** 10.3390/diagnostics13091646

**Published:** 2023-05-07

**Authors:** Sang Min Lee, Hong Il Ha, In Jae Lee, Kwanseop Lee, Jung Woo Lee, Ji Won Park, Sung-Eun Kim, Mi Jung Kwon, Ji-Young Choe, Sam-Youl Yoon, Seung-Gu Yeo, Min-Jeong Kim

**Affiliations:** 1 Department of Radiology, CHA University Gangnam Medical Center, Seoul 06135, Republic of Korea; 2 Department of Radiology, Hallym University Sacred Heart Hospital, Hallym University College of Medicine, Anyang 14068, Republic of Korea; 3 Department of Surgery, Hallym University Sacred Heart Hospital, Hallym University College of Medicine, Anyang 14068, Republic of Korea; 4 Department of Internal Medicine, Hallym University Sacred Heart Hospital, Hallym University College of Medicine, Anyang 14068, Republic of Korea; miunorijw@hallym.or.kr (J.W.P.);; 5 Department of Pathology, Hallym University Sacred Heart Hospital, Hallym University College of Medicine, Anyang 14068, Republic of Korea; 6 Anatomic Pathology Reference Lab, Seegene Medical Foundation, Seoul 04805, Republic of Korea; 7 Department of Surgery, Inje University Sanggye Paik Hospital, Seoul 01757, Republic of Korea; 8 Department of Radiation Oncology, Soonchunhyang University College of Medicine, Soonchunhyang University Hospital, Bucheon 14584, Republic of Korea

**Keywords:** two-dimensional shear wave elastography, point shear wave elastography, chronic liver disease, liver fibrosis, histology

## Abstract

Evaluation of hepatic fibrosis is essential to prevent liver-related morbidity and mortality. Although various types of ultrasound shear wave elastography (SWE) have been used and validated, there are limited studies on the relatively newer technique, two-dimensional SWE (2D-SWE). Therefore, this study aimed to compare the diagnostic performances of 2D-SWE and point SWE (p-SWE) for evaluating liver fibrosis using histology as the reference standard. To measure liver stiffness (LS) values, 87 patients underwent 2D-SWE and p-SWE using the same machine. Technical failures and unreliable measurements were also evaluated. The diagnostic performances of 2D-SWE and p-SWE were compared using area under the receiver operating characteristic (AUROC) curve analysis. No technical failures were observed in either method; however, unreliable measurements were less frequent in 2D-SWE (1/87 [1.1%]) than in p-SWE (8/87 [9.2%]) (*p* < 0.001). The AUROC of the LS values of 2D-SWE were significantly higher than those of p-SWE for diagnosing significant fibrosis (0.965 vs. 0.872, *p* = 0.022) and cirrhosis (0.994 vs. 0.886, *p* = 0.042). In conclusion, 2D-SWE is more reliable and accurate than p-SWE for diagnosing hepatic fibrosis.

## 1. Introduction

Chronic liver disease (CLD) is a major global health issue, responsible for two million liver-related deaths annually worldwide [1,2]. A wide range of etiologies, including viral infections (hepatitis B and C), toxins, alcohol abuse, autoimmune diseases, and genetic and metabolic disorders, can damage the liver parenchyma, leading to parenchymal regeneration with the recruitment of stellate cells and fibroblasts, and accumulation of excessive fibrous tissue in the liver [3,4,5]. Liver fibrosis, a principal consequence of CLD, can lead to portal hypertension, cirrhosis, liver failure, and hepatocellular carcinoma [5,6]. Therefore, a precise and timely diagnosis of hepatic fibrosis is crucial for assessing disease severity, guiding appropriate treatment decisions, monitoring disease progression over time, predicting clinical outcomes, and preventing liver-related morbidity and mortality [6,7,8]. Liver biopsy is considered as the standard method for assessing hepatic fibrosis. However, its routine use for monitoring is limited by its invasiveness, high cost, poor patient compliance, and limited accessibility [9,10]. Consequently, non-invasive tools for assessing hepatic fibrosis have emerged [10].

Ultrasound-based shear wave elastography (SWE) is an established noninvasive tool for staging liver fibrosis in patients with CLD [11,12,13,14,15,16]. This technique uses shear wave velocity to estimate tissue stiffness, which is correlated with the degree of fibrosis. Shear waves can be generated by external mechanical vibration (vibration-controlled transient elastography; TE) or by focused short-duration acoustic pulses enabling a single measurement (acoustic radiation force impulse imaging; ARFI imaging) or an image (supersonic shear wave imaging; SSI) [12,13,14]. In the United States, TE is commonly used to assess liver fibrosis [15]. Multiple studies have extensively validated TE and demonstrated its high diagnostic performance and reproducibility [13,14,16]. On the other hand, transient elastography (TE) has its drawbacks, including the inability to perform B-mode imaging and its high failure rates in individuals who are obese, have ascites, or have narrow intercostal spaces [17,18].

The ARFI imaging technique can be broadly grouped into two categories: point shear wave elastography (p-SWE) and two-dimensional shear wave elastography (2D-SWE), both of which allow simultaneous evaluation of liver morphology on gray-scale B-mode imaging and quantitative assessment of liver fibrosis using the same probe as that used in the conventional diagnostic ultrasound system [19]. p-SWE generates a single shear wave at a single frequency during each measurement, whereas 2D-SWE emits multiple shear waves at various frequencies throughout the examined area. Unlike p-SWE, which allows for a single region-of-interest (ROI) measurement, 2D-SWE enables the operator to select and analyze multiple circular ROIs (approximately 10 mm in diameter) within the larger ROI box [19,20]. Numerous studies have demonstrated that both p-SWE and 2D-SWE exhibit diagnostic accuracies comparable, or superior, to those of TE for measuring liver fibrosis [21,22,23,24]. However, limited available data suggest that 2D-SWE, a relatively newer technique, may have superior diagnostic accuracy compared with p-SWE [25,26,27]. Additionally, few published studies have validated the diagnostic performance of 2D-SWE for staging liver fibrosis using histology as the reference standard [28].

Therefore, the objective of this study was to compare the diagnostic performances of 2D-SWE and p-SWE, both equipped on the same machine, for the evaluation of liver fibrosis, using histology as the reference standard.

## 2. Materials and Methods

### 2.1. Study Population

**Figure 1:**
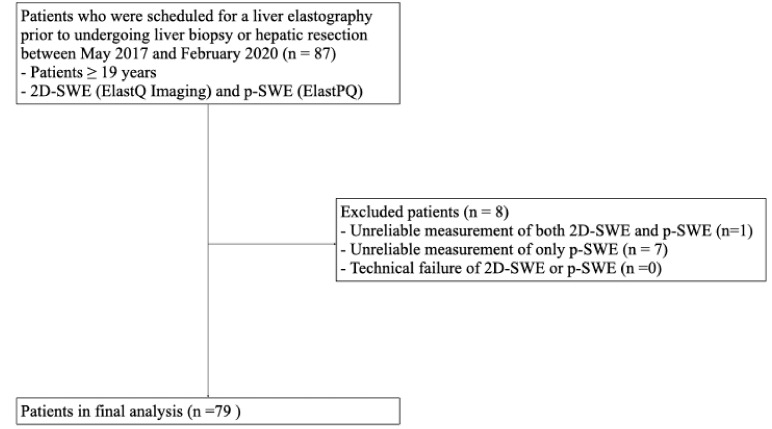
Flow diagram of the study population. 2D-SWE, two-dimensional shear wave elastography; p-SWE, point shear wave elastography.

This prospective pilot study was conducted with the approval of the Institutional Review Board (IRB) of Hallym University Sacred Heart Hospital (HALLYM 2017-I061), and written informed consent was obtained from all patients. Between May 2017 and February 2020, 87 patients who were scheduled to undergo a liver biopsy or hepatic resection were enrolled in this study (Figure 1). The inclusion criteria were as follows: (1) patients ≥ 19 years; (2) patients scheduled for liver parenchymal biopsy due to suspected chronic liver disease; (3) patients scheduled to undergo hepatectomy for various causes (e.g., hepatic tumor, transplantation, or liver donation).

### 2.2. Liver Stiffness Values Measurement Using 2D-SWE and p-SWE

**Figure 2:**
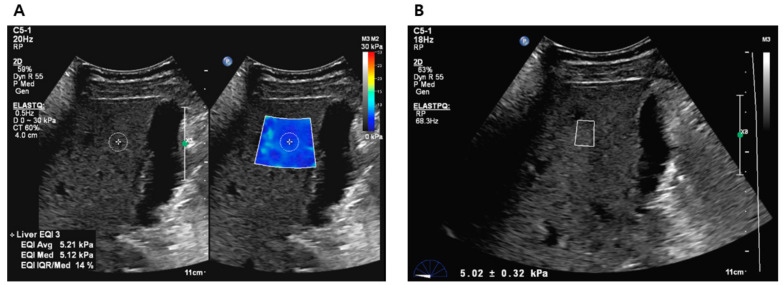
Liver stiffness value measurements using 2D-SWE (**A**) and p-SWE (**B**). 2D-SWE, two-dimensional shear wave elastography; p-SWE, point shear wave elastography.

All participants who provided consent were scheduled for liver elastography before undergoing liver biopsy or hepatic resection. The liver stiffness (LS) values were obtained using 2D-SWE (ElastQ Imaging) and p-SWE (ElastPQ) on the same ultrasound machine (EPIQ7G, Philips Healthcare, Cleveland, OH, USA). The elastography procedure was performed by an expert radiologist with >10 years of abdominal ultrasound experience and >5 years of SWE experience, using a convex probe (C5-1 probe) via an intercostal approach. Two sessions were conducted to measure LS values, with each session consisting of 10–15 sequential LS values measured using 2D-SWE and 10–15 sequential LS values measured using p-SWE. A B-mode scan was performed between the two sessions to avoid recall bias. Before the examination, patients were instructed to fast for at least 6 h and were positioned in a supine posture with their right arm elevated above the head. During the procedure, patients were instructed to briefly suspend their respiration (less than 5 s) [6,14]. For LS values measurement using 2D-SWE, a trapezoid-shaped, colored elastographic box was placed at a depth of 1.5–2.0 cm below and perpendicular to the liver capsule [25]. Two or three round ROI with a 1 cm diameter were drawn within the elastographic box (Figure 2A). For LS values measurement using p-SWE, a square ROI (1.5 cm × 0.5 cm) was positioned in the same position as the liver (Figure 2B). The ROIs were placed to avoid hepatic vessels and masses.

Technical failure of 2D-SWE was defined as a colored filling of <50% of the elastographic box in all measurements [29]. For p-SWE, failure to obtain 10 valid values during 10–15 sequential measurements was considered a technical failure. Unreliable measurements were defined as an interquartile range (IQR)/median ratio of LS > 30% [6]. The representative LS value for each session was the median LS measurement.

### 2.3. Clinical Data and Biomarkers for Fibrosis

Clinical data, including age, sex, weight, body mass index (BMI), aspartate aminotransferase (AST), alanine aminotransferase (ALT), and platelets were obtained from the electronic medical records (EMR) of the institution, which were collected within one week prior to performing US elastography. Based on clinical data, biomarkers for hepatic fibrosis were derived. The AST-to-platelet ratio index (APRI) was defined as (AST/upper limit of normal)/platelet count (10^9^/L) × 100. The fibrosis index based on four factors (FIB-4) was defined as [age (years) × AST (IU/L)]/[platelets (10^9^/L) × ALT^1/2^ (IU/L)].

### 2.4. Histologic Analysis

The specimens were fixed in a formalin–alcohol–acetic acid solution, embedded in paraffin, cut, and stained with hematoxylin and eosin. Two expert pathologists (with >10 years of experience in hepatic pathology) without knowledge of the clinical data and LS values independently analyzed the specimens based on the METAVIR scores. In case of disagreement, a consensus was reached between the pathologists to determine the final scoring. Fibrosis was classified as no fibrosis (F0), portal fibrosis (F1), periportal fibrosis (F2), septal fibrosis (F3), or cirrhosis (F4). The necroinflammatory activity grade, consisting of porto-periportal and lobular activity, was classified as no activity (A0), minimal activity (A1), mild activity (A2), moderate activity (A3), and severe activity (A4). Steatosis was graded from S0 to S4 (S0: absent steatosis; S1: <5%; S2: 5–33%; S3: 33–66%; and S4: >66%).

### 2.5. Statistical Analysis

Statistical analyses were performed using MedCalc (version 20.218; MedCalc software, Mariakerke, Belgium) and SPSS software (version 27.0; SPSS, Inc., Chicago, IL, USA). To compare continuous values (e.g., LS values of 2D-SWE and p-SWE), a paired *t*-test or Wilcoxon test was used. Fisher’s exact test or chi-square test was used to compare categorical values. Spearman’s correlation test was used to determine the correlation between METAVIR scores (fibrosis, steatosis, and necroinflammation staging) and LS values. Subsequently, multiple regression analysis was used to determine the independent factors affecting the LS values.

The diagnostic performances of 2D-SWE and p-SWE were evaluated and compared using area under the receiver operating characteristic (AUROC) curve analysis based on histological staging as a reference standard. The optimal cutoff values for diagnosing significant fibrosis (≥F2) and cirrhosis (F4) were obtained using Youden index. A *p*-value < 0.05 was considered statistically significant.

## 3. Results

### 3.1. Technical Failure and Unreliable Measurement

Of the 87 patients enrolled in this study, eight were excluded from the final analysis because of unreliable 2D-SWE and p-SWE measurements. Unreliable measurements were significantly less frequent with 2D-SWE (1/87 [1.1%]) than with p-SWE (8/87 [9.2%]) (*p* < 0.001). No technical failures were observed in either 2D-SWE or p-SWE. Therefore, 79 patients with reliable measurements obtained using both SWE methods were included in the final analysis (Figure 1).

### 3.2. Clinical and Histologic Features in Patients Included in the Final Analysis

The 79 patients in the final analysis consisted of 4 patients who underwent liver biopsy and 75 patients who underwent hepatic resection. Histological confirmation was performed within 1 week after LS measurements, with a median interval of 1 day (range: 0–7 days). The histologic diagnosis of 79 patients included hepatocellular carcinoma (*n* = 26), cholangiocarcinoma (*n* = 23), combined hepatocellular-cholangiocarcinoma (*n* = 10), hepatic metastasis (*n* = 10), necrotic nodules associated with transarterial chemoembolization (*n* = 2), hepatolithiasis (*n* = 2), intraductal papillary neoplasm of bile duct (*n* = 2), alcoholic liver cirrhosis (*n* = 1), primary biliary cirrhosis (*n* = 1), simple steatosis (*n* = 1), and normal parenchyma without steatohepatitis (*n* = 1). The clinical characteristics and METAVIR scores of the 79 patients (51 men, 28 women; mean age, 62.2 years ± 11.1 [standard deviation]) are presented in Table 1. Significant fibrosis (≥F2) was observed in 29 (36.7%) of 79 patients and cirrhosis (F4) was observed in 17 (21.5%) of 79 patients.

### 3.3. Correlation with Liver Stiffness Values of 2D-SWE and p-SWE and METAVIR Scores

**Figure 3:**
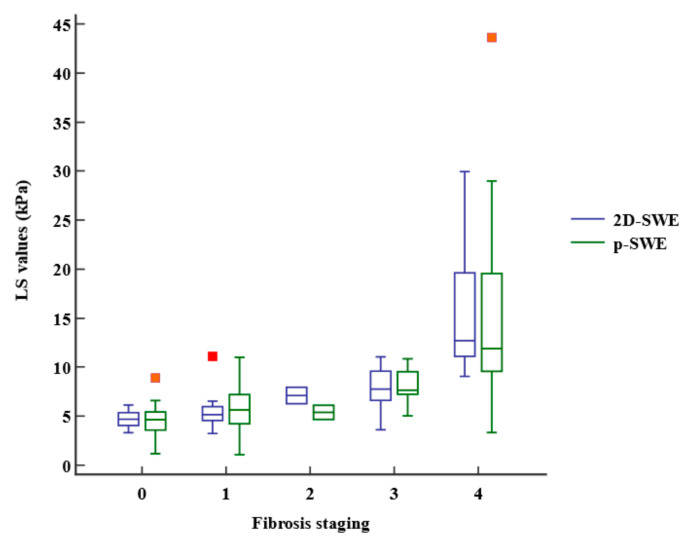
Box-and-whisker plot of the liver stiffness (LS) values at each fibrosis staging. The boxes indicate the LS values between the 25th and 75th quartiles, and the lines at the center of the boxes represent the median. The whiskers represent the 9th and 91st percentiles. Small orange squares represent outlier LS values measured using p-SWE and red orange square represents outlier LS value measured using 2D-SWE. LS, liver stiffness; 2D-SWE, two-dimensional shear wave elastography; p-SWE, point shear wave elastography.

The LS values obtained using 2D-SWE and p-SWE increased as the fibrosis stage increased (Figure 3). However, there was no significant difference in the median LS values measured using 2D-SWE and p-SWE for each fibrosis stage (Table 2). The LS values of 2D-SWE and METAVIR fibrosis stages exhibited a strong positive correlation (r = 0.762, 95% confidence interval [CI]: 0.651–0.841, *p* < 0.001), and those of p-SWE also showed a high correlation with the fibrosis stages (r = 0.652, 95% CI: 0.502–0.764, *p* < 0.001) (Table 3). However, the LS values on 2D-SWE showed a weak correlation with necroinflammation in the METAVIR score (r = 0.270, 95% CI: 0.050–0.464, *p* = 0.017). The LS values on p-SWE did not correlate with necroinflammation. The steatosis and LS values on 2D-SWE and p-SWE were not correlated. Multiple regression analysis revealed that fibrosis staging only (β = 1.77, *p* < 0.001, for 2D-SWE; β = 1.53, *p* < 0.001, for p-SWE) was an independent factor affecting LS values of 2D-SWE (R^2^ = 0.613, *p* < 0.001) and those of p-SWE (R^2^ = 0.443, *p* < 0.001).

### 3.4. Comparison of Diagnostic Performance between 2D-SWE and p-SWE

**Figure 4:**
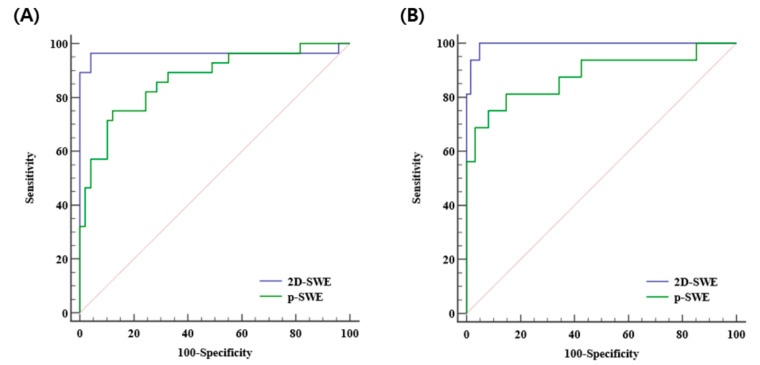
Area under the receiver operating curves of 2D-SWE and p-SWE in diagnosing significant fibrosis (≥F2) (**A**) and cirrhosis (F4) (**B**). 2D-SWE, two-dimensional shear wave elastography; p-SWE, point shear wave elastography.

The diagnostic performances of 2D-SWE and p-SWE for diagnosing significant fibrosis (≥F2) were excellent with the AUROC of 0.965 (95% CI: 0.895–0.993) and 0.872 (95% CI: 0.777–0.937), respectively. The optimal cutoff values for 2D-SWE and p-SWE for significant fibrosis were 6.26 kPa and 7.08 kPa, respectively. For diagnosing cirrhosis (F4), the diagnostic performances of both 2D-SWE and p-SWE were excellent, with AUROC of 0.994 (95% CI: 0.943–1.00) and 0.886 (95% CI: 0.794–0.947), respectively. The optimal cutoff values for 2D-SWE and p-SWE for cirrhosis were 8.40 kPa and 9.30 kPa, respectively (Table 4). The AUROC of 2D-SWE was significantly higher than that of p-SWE for diagnosing significant fibrosis (0.965 vs. 0.872, *p* = 0.022) and cirrhosis (0.994 vs. 0.886, *p* = 0.042) (Figure 4).

The diagnostic performance of APRI and FIB-4 for detecting significant fibrosis was suboptimal with AUROCs of 0.6 (95% CI: 0.482–0.710) and 0.637 (95% CI: 0.520–0.744) and was also suboptimal for cirrhosis with AUROCs of 0.655 (95% CI: 0.538–0.759) and 0.688 (95% CI: 0.572–0.788), respectively (Supplementary Table S1). 2D-SWE showed the best diagnostic performance, followed by p-SWE, APRI, and FIB-4 in distinguishing between significant fibrosis and cirrhosis (Supplementary Figure S1).

## 4. Discussion

We prospectively conducted this study to directly compare the diagnostic performances of two newer SWE techniques, 2D-SWE (ElastQ imaging) and p-SWE (ElastPQ), on the same machine (EPIQ7G, Philips Healthcare) for detecting liver fibrosis, using the histological staging of hepatic fibrosis as a reference standard. Our results demonstrated that the LS values of 2D-SWE were strongly correlated with histologic fibrosis stage (r = 0.762), and those of p-SWE also showed a high correlation with fibrosis stage (r =0.652). 2D-SWE exhibited better diagnostic performance than p-SWE for detecting significant fibrosis, with excellent AUROCs of 0.965 and 0.872, and for cirrhosis, with AUROCs of 0.994 and 0.886, respectively.

Numerous studies have reported a significant correlation between liver fibrosis stages based on histology and LS values obtained using p-SWE, which has demonstrated comparable or better diagnostic performance than TE [21,23,24,25]. In contrast, 2D-SWE is a relatively newer technique with limited available data, based mostly on the supersonic shear imaging (Aixplorer^®^, Supersonic Imagine, Aix-en-Provence, France). Several studies have shown that LS values obtained using supersonic shear imaging are strongly correlated with the fibrosis stage based on histology. Furthermore, it has demonstrated comparable or better diagnostic accuracy than p-SWE and TE in discriminating between significant fibrosis and cirrhosis, using liver biopsy as the reference standard [22,23,26,28,29]. However, ElastQ imaging, the latest 2D-SWE technique used in our study, has not yet been extensively validated. A recent meta-analysis, which included 23 articles on 2D-SWE and 48 articles on p-SWE, revealed that 2D-SWE had a higher diagnostic performance than p-SWE for detecting significant fibrosis with AUROCs of 0.89 and 0.85 and for cirrhosis with AUROCs of 0.94 and 0.91, respectively [30]. Although the meta-analysis yielded results similar to those of our study, none of the studies in the meta-analysis directly compared 2D-SWE and p-SWE. In contrast, our study directly compared the diagnostic performances of 2D-SWE and p-SWE, which were performed on the same machine and used in the same population. Since all variables (e.g., US machine, US probe, patient, operator) except for the SWE techniques were the same, our study could exactly reflect the influence of the SWE technique on the diagnostic performance for the evaluation of liver fibrosis. Furthermore, unlike other studies, our study compared the diagnostic performances of 2D-SWE and p-SWE using histological confirmation as a reference standard [25,31]. Additionally, to the best of our knowledge, previous studies have mostly used supersonic shear imaging as a 2D-SWE machine, and there were few studies using the ElastQ imaging used in our study.

Here, both 2D-SWE and p-SWE showed much higher diagnostic performance for the detection of significant fibrosis and cirrhosis than biomarkers (APRI and FIB-4). In contrast, other studies reported no statistical difference in the diagnostic performance of transient elastography for cirrhosis among SWE, APRI, and FIB-4 [32]. A meta-analysis comparing p-SWE, APRI, and FIB-4 reported that, similar to our results, p-SWE showed better diagnostic performance than biomarkers [33].

Our results demonstrated that among METAVIR scores (fibrosis, necroinflammation, steatosis), fibrosis staging was an independent factor affecting LS values in both 2D-SWE and p-SWE. This finding is similar to those of previous studies [29,34]. In a study comparing TE and 2D-SWE in patients with non-alcoholic fatty liver disease, necroinflammation affected LS values measured by TE but not by 2D-SWE [34]. A previous study comparing the diagnostic accuracy of 2D-SWE, p-SWE, and TE also reported that all three techniques correlated with the degree of fibrosis and necroinflammation, but not steatosis [23]. In our study, the LS values on 2D-SWE showed a weak correlation with necroinflammation on the METAVIR score. Therefore, we hypothesized that necroinflammation could have some impact on LS values measured using SWE.

Our study found that 2D-SWE had fewer unreliable measurements than p-SWE, which is consistent with the findings of previous studies [25,28]. This may be due to the larger elastographic box with color coding in 2D-SWE, which allows for more appropriate placement of ROIs away from the artifact areas. In addition, 2D-SWE requires fewer acquisitions than p-SWE [6]. Another study using the latest 2D-SWE technique, ElastQ Imaging, reported that 2D-SWE has technical advantages over p-SWE, including greater reliability and speed [25]. However, this study did not evaluate the diagnostic performance of these two techniques. Our prospective study directly compared the diagnostic performance of 2D-SWE and p-SWE, using histology as a reference standard.

This study has several limitations. First, the number of patients included was small, and this prospective study was conducted at a single center. Second, our patients were not equally distributed according to liver fibrosis stage. Only two patients had an F2 score, and although this limited number could potentially impact the interpretation of our study, our focus was on discriminating significant fibrosis (≥F2) and cirrhosis (F4) rather than evaluating diagnostic performance for each fibrosis stage separately. Thus, we believe the impact of the limited number of F2 patients on our study results was negligible. Third, the etiologies of the underlying liver disease were heterogeneous. Cutoff values are affected by the etiology of the underlying liver disease [35]. However, it is not feasible to recruit patients with a single disease in a clinical setting, and many previous studies targeted patients with various causes of chronic liver disease. Fourth, patients with hepatic tumors were included in this study. While previous studies have also included patients with hepatic tumors to evaluate hepatic fibrosis and have placed ROIs to avoid hepatic tumors [29,36,37,38], it is possible that the presence of a tumor could still affected the liver stiffness values. However, in our study, we took care to place ROIs in tumor-free areas to minimize the potential impact of tumors on liver stiffness measurements. Finally, only one radiologist measured the LS values using 2D-SWE and p-SWE without evaluating the intra- and inter-observer variability. Our study measured the LS values using two techniques on the same machine and within the same population, which provides a more valid way to compare different tests. In addition, a recent meta-analysis reported that 2D-SWE had good-to-excellent intra-observer reliability (ICC = 0.93) and inter-observer reliability (ICC = 0.87) [27]. Further large-scale multicenter studies with the same etiology of underlying liver disease are needed to evaluate the diagnostic performance and increase the level of evidence.

## 5. Conclusions

For the detection of hepatic fibrosis, 2D-SWE and p-SWE are good noninvasive tools with excellent diagnostic performance, particularly for discriminating significant fibrosis and cirrhosis. In addition, 2D-SWE has better diagnostic performance than p-SWE, with greater reliability. Serologic biomarkers, including APRI and FIB-4, showed lower diagnostic accuracy than SWE. Thus, 2D-SWE may be a suitable alternative to liver biopsy for the diagnosis and monitoring of hepatic fibrosis in clinical practice.

## Figures and Tables

**Table 1:** Clinical features and METAVIR scores of 79 patients in the final analysis.

Mean Age (Years)	62.2 ± 11.1
Sex	
Male	51 (64.6%)
Female	28 (35.4%)
Body mass index (kg/m^2^)	24.5 ± 3.6
Laboratory data	
Aspartate transaminase (U/L)	121.2 ± 220.4
Alanine transaminase (U/L)	83.5 ± 102.4
Alkaline phosphatase (U/L)	133.1 ± 113.2
ɣ-Glutamyl transferase (U/L)	166.7 ± 343.0
Total bilirubin (µmol/L)	2.1 ± 3.7
Prothrombin time (s)	14.5 ± 2.1
Albumin (g/dL)	3.7 ± 0.2
Platelet count (10^9^/L)	195.3 ± 92.7
APRI	2.1 ± 4.0
FIB-4	4.9 ± 7.5
Underlying liver disease	
Chronic hepatitis B	32 (40.5%)
Chronic hepatitis C	6 (7.6%)
Chronic alcohol hepatitis	5 (6.3%)
Idiopathic chronic hepatitis	4 (5.1%)
Nonalcoholic steatohepatitis	3 (3.8%)
Autoimmune hepatitis	1 (1.3%)
None	28 (35.4%)
METAVIR Scores	
Fibrosis	
F0	27 (34.2%)
F1	23 (29.1%)
F2	2 (2.5%)
F3	10 (12.7%)
F4	17 (21.5%)
Steatosis	
S0	20 (25.3%)
S1	15 (19.0%)
S2	25 (31.6%)
S3	13 (16.5%)
S4	6 (7.6%)
Necroinflammation	
A0	22 (27.8%)
A1	15 (19.0%)
A2	18 (22.8%)
A3	24 (30.4%)

APRI, aspartate aminotransferase to platelet ratio index; FIB-4, fibrosis index based on four factors. Values are presented as means ± standard deviations or numbers with percentages.

**Table 2:** LS values obtained using 2D-SWE and p-SWE on each fibrosis stage.

	LS Values Using 2D-SWE	LS Values Using p-SWE	*p*-Values
All	5.71 (4.60, 8.56)	5.98 (4.43, 8.83)	0.427
F0	4.69 (4.05, 5.36)	4.65 (3.59, 5.43)	0.589
F1	5.16 (4.56, 5.97)	5.65 (4.23, 7.21)	0.540
F2	7.11 (NA)	5.39 (NA)	NA
F3	7.77 (6.62, 9.60)	7.66 (7.24, 9.54)	1.000
F4	12.75 (11.28, 19.67)	12.23 (9.04, 19.95)	0.151

LS, liver stiffness; 2D-SWE, two-dimensional shear wave elastography; p-SWE, point shear wave elastography; NA, not available. The *p*-values were calculated using Wilcoxon test. Values are presented as median (interquartile range).

**Table 3:** Correlation of METAVIR scores and liver stiffness values obtained using 2D-SWE and p-SWE.

		Correlation Coefficient, r	95% CI for r	*p*-Values
Fibrosis	2D-SWE	0.762	0.651, 0.841	<0.001
	p-SWE	0.652	0.502, 0.764	<0.001
Necroinflammation	2D-SWE	0.27	0.050, 0.464	0.017
	p-SWE	0.139	−0.088, 0.352	0.230
Steatosis	2D-SWE	0.255	0.011, 0.487	0.060
	p-SWE	0.081	−0.188, 0.339	0.556

CI, confidence interval; 2D-SWE, two-dimensional shear wave elastography; p-SWE, point shear wave elastography.

**Table 4:** Comparison of diagnostic performance of 2D-SWE and p-SWE for significant fibrosis (≥F2) and cirrhosis (F4).

	≥F2			F4		
	2D-SWE	p-SWE	*p*-Value	2D-SWE	p-SWE	*p*-Value
AUROC	0.965	0.872	0.022	0.994	0.886	0.042
95% CI	0.895, 0.993	0.777, 0.937		0.943, 1.00	0.794, 0.947	
optimal cutoff value (kPa)	6.26	7.08		8.4	9.3	
sensitivity (%)	96.7	75.9		100	76.5	
specificity (%)	95.9	87.8		95.1	91.8	

AUROC, area under the receiver operating curve; CI, confidence interval; 2D-SWE, two-dimensional shear wave elastography; p-SWE, point shear wave elastography.

## Data Availability

No new data were created or analyzed in this study. Data sharing is not applicable to this article.

## Supplementary Materials

The following supporting information can be downloaded at: https://www.mdpi.com/article/10.3390/diagnostics13091646/s1, Figure S1. Area under the receiver operating curves of 2D-SWE, p-SWE, APRI, and FIB-4 in diagnosing significant fibrosis (≥F2) (A) and cirrhosis (F4) (B), 2D-SWE, two-dimensional shear wave elastography; p-SWE, point shear wave elastography; APRI, aspartate aminotransferase to platelet ratio index, FIB-4 = fibrosis index based on four factors; Table S1. Comparison of diagnostic performances of 2D-SWE, p-SWE, and biomarkers for diagnosis of significant fibrosis (≥F2); Table S2. Comparison of diagnostic performances of 2D-SWE, p-SWE, and biomarkers for diagnosis of cirrhosis (F4).
