# Supplementary material for: Comparison between Two-Dimensional and Point Shear Wave Elastography Techniques in Evaluating Liver Fibrosis Using Histological Staging as the Reference Standard: A Prospective Pilot Study
Source: Diagnostics (Basel). 2023 May 7;13(9):1646. doi: 10.3390/diagnostics13091646 (PMC10178401; doi:10.3390/diagnostics13091646)
Supplement: Supplementary file 1 [file diagnostics-13-01646-s001.zip › diagnostics-2347915-supplementary.pdf]

Supplementary Materials:

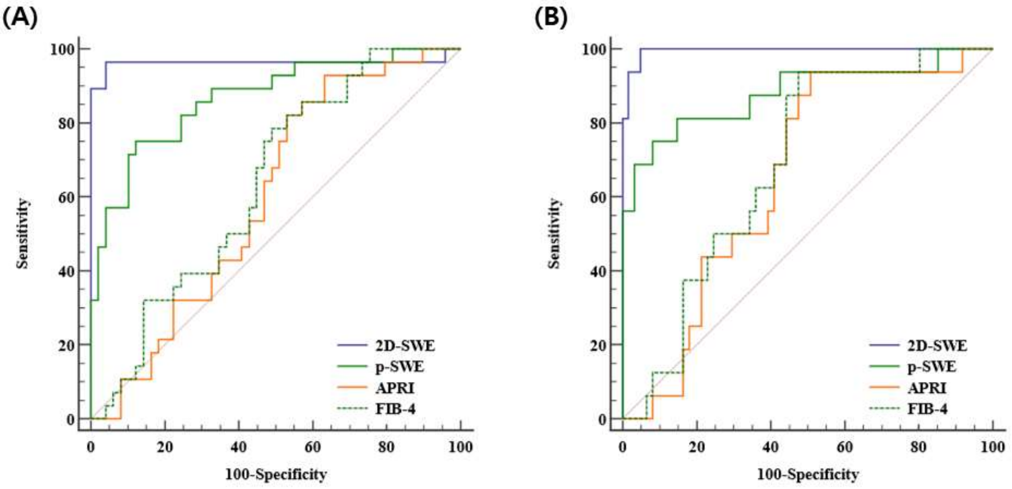

Figure S1. Area under the receiver operating curves of 2D-SWE, p-SWE, APRI, and FIB-4 in diagnosing significant fibrosis (≥F2) (**A**) and cirrhosis (F4) (**B**), 2D-SWE, two-dimensional shear wave elastography; p-SWE, point shear wave elastography; APRI, aspartate aminotransferase to platelet ratio index, FIB-4 = fibrosis index based on four factors

Table S1. Comparison of diagnostic performances of 2D-SWE, p-SWE, and biomarkers for diagnosis of significant fibrosis (≥F2)

	**Significant Fibrosis (≥F2)**				***p*-Values**					
	**2D-SWE [a]**	**p-SWE [b]**	**APRI [c]**	**FIB-4 [d]**	**a vs. b**	**a vs. c**	**a vs. d**	**b vs. c**	**b vs. d**	**c vs. d**
AUROC	0.965	0.872	0.6	0.637	0.022	<0.001	<0.001	<0.001	0.001	0.19
95% CI	0.895, 0.993	0.777, 0.937	0.482, 0.710	0.520, 0.744						
optimal cutoff value	6.26 kPa	7.08 kPa	0.472	2.33						
sensitivity (%)	96.7	75.9	83.3	80						
specificity (%)	95.9	87.8	46.9	51						

AUROC, area under the receiver operating curve; CI, confidence interval; 2D-SWE [a], two-dimensional shear wave elastography; p-SWE [b], point shear wave elastography; APRI [c], aspartate aminotransferase to platelet ratio index; FIB-4 [d], fibrosis index based on four factors.

Table S2. Comparison of diagnostic performances of 2D-SWE, p-SWE, and biomarkers for diagnosis of cirrhosis (F4)

	**Cirrhosis (F4)**				***p*-Values**					
	**2D-SWE [a]**	**p-SWE [b]**	**APRI [c]**	**FIB-4 [d]**	**a vs. b**	**a vs. c**	**a vs. d**	**b vs. c**	**b vs. d**	**c vs. d**
AUROC	0.994	0.886	0.655	0.688	0.041	<0.001	<0.001	0.006	0.017	0.403
95% CI	0.943,1.00	0.794, 0.947	0.538, 0.759	0.572, 0.788						
optimal cutoff value	8.4 kPa	9.3 kPa	0.597	2.37						
sensitivity (%)	100	76.5	94.4	94.4						
specificity (%)	95.1	91.8	49.2	52.5						

AUROC, area under the receiver operating curve; CI, confidence interval; 2D-SWE [a], two-dimensional shear wave elastography; p-SWE [b], point shear wave elastography; APRI [c], aspartate aminotransferase to platelet ratio index; FIB-4 [d], fibrosis index based on four factors.
